# Simultaneous determination of low molecule benzotriazoles and benzotriazole UV stabilizers in wastewater by ultrasound-assisted emulsification microextraction followed by GC–MS detection

**DOI:** 10.1038/s41598-021-89529-1

**Published:** 2021-05-12

**Authors:** Urszula Kotowska, Joanna Struk-Sokołowska, Janina Piekutin

**Affiliations:** 1 Department of Analytical and Inorganic Chemistry, Faculty of Chemistry, University of Bialystok, Ciołkowskiego 1K Street, 15-245 Białystok, Poland; 2 Department of Environmental Engineering Technology, Faculty of Civil Engineering and Environmental Sciences, Bialystok University of Technology, Wiejska 45E, 15-351 Białystok, Poland

**Keywords:** Analytical chemistry, Green chemistry, Environmental chemistry

## Abstract

A rapid, sensitive, economically and ecologically friendly method based on one-step ultrasound-assisted emulsification microextraction and in situ derivatization followed by gas chromatography–mass spectrometry for simultaneous determination of low molecular benzotriazoles and benzotriazole-based ultraviolet filters was developed. The optimized method allows quantification of benzotriazole, 4-methylbenzotriazole, 5-methylbenzotriazole; 5-chlorobenzotriazole, 2-(2′-hydroxy-3′-tert-butyl-5′-methylphenyl)-5-chlorobenzortriazole and 2-(2′-hydroxy-5′-(1,1,3,3-tetramethylbutyl)phenyl)benzotriazole in municipal and industrial (dairy) wastewater. The method was validated using real influent and effluent wastewater and samples at various stages of the purification process. Relative recoveries obtained using wastewater as sample matrix were between 77 and 137%, method limits of detection from 0.001 to 0.035 µg/L, method limits of quantification from 0.003 to 0.116 µg/L, the repeatability expressed by the coefficient of variation did not exceed 12%. The use of the method for the determination of tested compounds in municipal and industrial wastewater showed their presence in most of the tested samples, in concentrations from LoD to 6.110 µg/L. The conducted studies of samples from municipal wastewater treatment plant located in north-east Poland showed that the effectiveness of benzotriazole removal by this plant wasfrom 29 to 84%. The load of tested compounds released into the environment by this facility ranges from 2 to 269 mg/day/1000 inhabitants.

## Introduction

The presence of organic micropollutants in various elements of the environment is a major problem as these compounds pose a significant threat to living organisms or entire ecosystems while the presence is caused by rapid industrial and technological development^1^. Low molecule benzotriazoles (LMBTs) are polar compounds and have good solubility in water^2–4^. It is easily introduced into other chemical structures through a number of reactions such as condensation or addition^5^. Their properties also include considerable resistance to oxidation and biodegradation^6^. BTs are only partialy removed by activated sludge and other treatment processes such as chlorination and advanced oxidation^7^.

BTs containing in their structure a hydrogen atom in position 1 and compounds with methyl group, especially 5-methylbenzotriazole (5MBT) and 4-methylbenzotriazole (4MBT) have anti-corrosion properties. BTs can be used as solder, brass, steel, cast iron and aluminium protection agents and are used in heating systems^3^. Phenyl group of BTs, especially in position 2, absorb UV light in the 300–400 nm wavelength range. There is a large group of compounds containing a BT moiety used to stabilise against UV radiation^8^. BT-based ultraviolet filters (BUVs) protect against degradation and yellowing of products. They are used in the production of paints, films, plastics, coatings and skin care products^9,10^. BT and its derivatives are a component of detergents, including dishwasher tablets and powder as well as a component of antifreeze agents and de-icing agents. Antifreeze mixtures for various surfaces (roads, airports, car parks) are widespread and widely used and as a result the risk of BTs entering the environment is very high^11^. BT derivatives are widely used in the pharmaceutical industry, petroleum products and one of the main objectives of using these compounds is to increase the efficiency and durability of products^2^.

The biological activity of many BT derivatives and their durability in the environment is connected with observed negative impact on the aquatic and terrestrial organisms^12^. These compounds are classified as hazardous to the environment and cause long-term harmful effects^13^. BTs can be absorbed by inhaling the aerosol and by ingestion^14^. Toxicological studies have shown that BTs have mutagenic and toxic effects on some microorganisms including algae and plants^15,16^. BTs can inhibit the growth of bacteria and weaken their ability to degrade certain organic compounds leading to impairment of self-purification process when these compounds enter rivers from wastewater treatment plants^4^. The BTs have been shown to be genotoxic and may disrupt hormonal management^17^. BT at higher concentrations show antagonistic interaction with the androgenic receptor and potential disruption of the thyroid hormone system and fatty acid metabolism^18^. It has been proven that 1HBT has carcinogenic properties for humans. According to Shi et al. BTs are associated with the development of endometrial cancer^4^. Toxicity studies have shown that direct contact with some widely used BUVs can cause inflammation and skin irritation. Gender-related hematological and histopathological changes in the liver, kidneys, spleen and thyroid glands were observed after long-term toxicity testing of 2-(3,5-di-tert-butyl-2-hydroxyphenyl) benzotriazole (UV-320) in rats. Similar properties were also shown for 2-(3,5-di-tertert-butyl-2-hydroxyphenyl) benzotriazole (UV-328)^19^. Some BUVs were also found to have similar estrogenic effects to those shown by the natural sex hormone 17-β-estradiol^20^.

BTs are the fourth most abundant aquatic contaminants after ethylenediamine tetraacetic acid and its salts, nitrilotriacetic acid alkylbenzene sulfonates^21^ and their annual consumption in the United States alone exceeds 9000 tonnes and is very high in all developed economies^22,23^. Quantification of BTs and its derivatives in environmental samples is carried out mainly by chromatographic methods although literature describes the use of electrochemical and spectrophotometric methods for this purpose^24,25^. High-performance or ultra-high-performance liquid chromatography (HPLC, UHPLC) is usually used to determine BTs in the environmental and in biological samples. Detection is carried out using UV spectrophotometr, diode array detector (DAD) or mass spectrometer (MS)^19,26–29^. The greatest sensitivity and selectivity of the determinations was achieved using UHPLC with tandem MS detector of triple quadrupole (QqQ MS/MS) or electrospray ionization (ESI MS/MS) configuration^30^. Determination of highly polar low mass compounds from the group of BT derivatives by gas chromatography (GC) requires the use of polar stationary phases or the conversion of compounds to less polar and more volatile derivatives which can be achieved by by acetylation or methylation^6,13,20,31–33^. Less polar BT derivatives such as BUVSs can be analyzed by GC on standard medium polar columns without a derivatization process^22,34^. MSs with electron ionization (EI) and a quadrupole (Q) or a time-of-flight (ToF) analyzers as well as tandem mass spectrometers (MS/MS) were used as GC detectors. Undoubtedly the isolation and enrichment of analytes is a key step in the correct analytical procedures for the determination of trace contaminants in a complex matrix. Wastewaters contain many chemical compounds often with similar properties and with a large variation in composition over time. A solid phase extraction (SPE) technique is usually used to extract benzotriazoles from aqueous samples^16,28,35^. In recent years microextraction techniques have become more and more important in environmental analysis^36,37^. Ahmad et al. described the use of bar adsorptive microextraction (BAME) to isolate BT and 5MBT from rain, tap, estuarine waters as well as wastewater^29^. Dispersive liquid–liquid microextraction (DLLME) was used for isolation of low molecule benzotriazoles in water samples^33^. Lu et al. proposed the use of air assisted liquid–liquid microextraction (AALLME) in the procedure for the determination of BT, 5MBT and 5-chlorobenzotriazole (5ClBT) in aqueous samples^26^.

The aim of the presented work was to develop a simple methodology based on ultrasound assisted emulsification microextraction (USAEME) with in-situ derivatization and GC–MS for simultaneous determination four LMBTs: BT, 4MBT, 5MBT, and 5ClBT as well as two BUVs: UV326 and UV329 in wastewater. The USAEME technique described for the first time by Requeiro et al. is an isolation technique based on the emulsification of a microliter volume of organic solvent in aquous sample by ultrasound radiation and separation of both phases by centrifugation^38^. Literature review shows that so far USAEME has not been used for determination of BTs in any matrix and no liquid–liquid microextraction has ever been used in the determination of the BUVs in any samples. Samples taken from municipal wastewater treatment plants (WWTPs) collected at various stages of the technological process and wastewater from Milk Processing Plant (MPP) have been analyzed. The main source of BTs in both matrices are household and industrial detergents. Wastewater from MPP after initial treatment is usually directed to municipal WWTPs. Determining the concentration of BTs in these matrices is important due to their influence on treatment processes (potential harmful effect on activated sludge). It will also allow to assess the exposure of aquatic environment to these compounds in connection with the discharge of wastewater into the environment after treatment process.

## Results and discussion

### Optimization of the extraction and derivatization procedure

**Figure 1:**
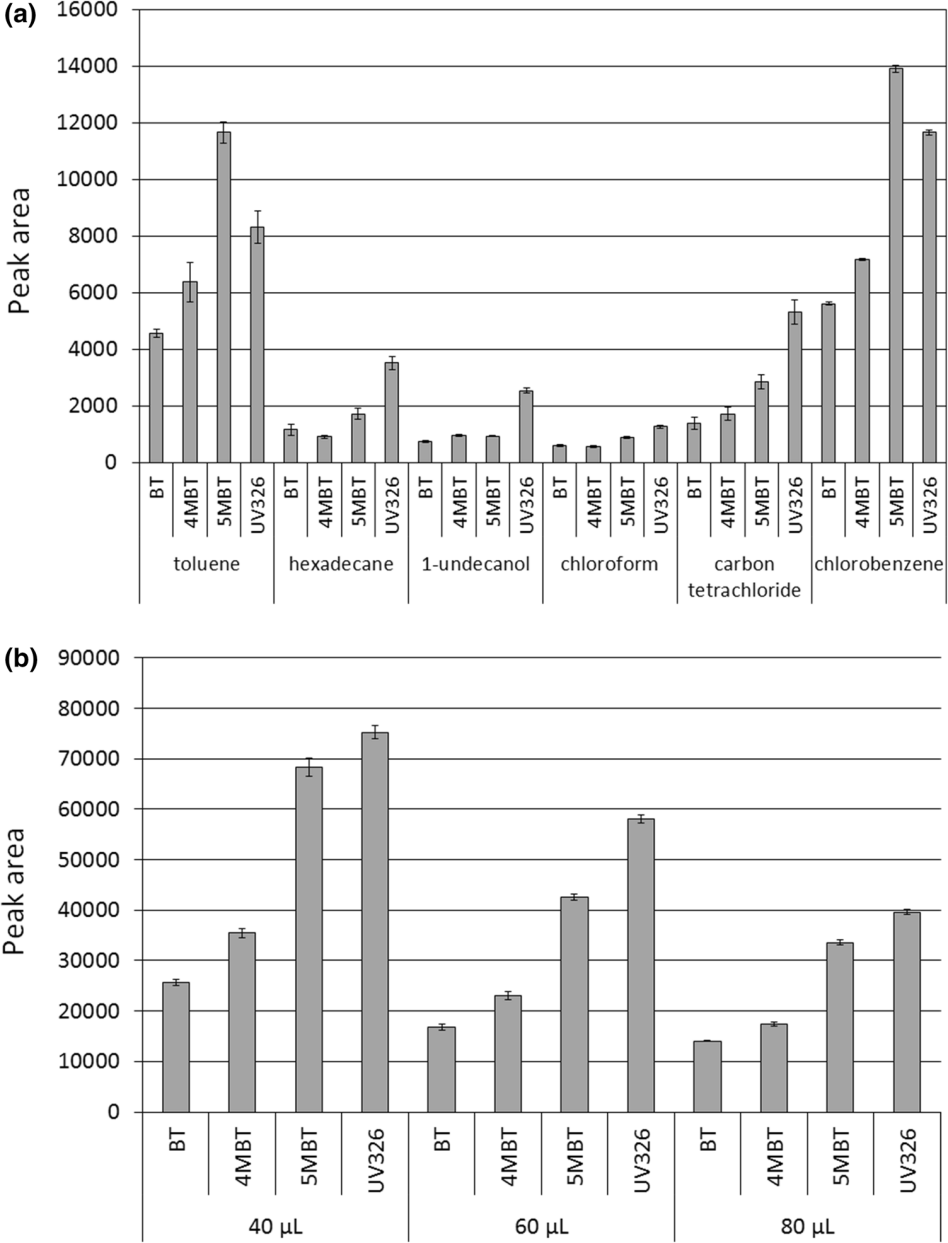
Influence of the type of solvent (**a**) and the volume of chlorobenzene (**b**) on the efficiency of benzotriazole extraction by the USAEME technique.

Very few procedures for the determination of benzotriazoles by GC in combination with single quadrupole MS have been published^23,31,33,35,39^. Based on previous literature reportsan acetylation reaction was selected for the derivatization of BTs in this work carried out simultaneously with the extraction process. A careful impact analysis has been carried out for the following process conditions type and volume of the extraction solvent, volume of acetic anhydride (derivatization reagent), addition of a buffer salt and time of simultaneous extraction and derivatization. *n*-Hexadecane, 1-undecanol, chloroform, carbon tetrachloride, toluene and chlorobenzene were tested as potential extraction solvents. The characteristics of selected solvents are included in Supplementary Table S1 (Supplementary Information). The responses obtained for each analyzed compound with different solvents used for extraction are shown in Fig. 1a. The volume of each of the solvents used for extraction was 100 μL. Optimization was performed for milli-Q water to which a mixture of analytes was introduced with a concentration of 10 μg/L each. Other conditions of the process were 100 μL of acetic anhydride and five minutes extraction time; the same conditions were used in all optimization experiments unless otherwise stated.

Chlorinated solvents with a density greater than that of water were selected for testing (see Supplementary Table S1, Supplementary Information), enabling easy removal of the organic layer after extraction by using test tubes with a conical bottom^38^. *n*-Hexadecane and 1-undecanol can be from the aqueous solution after extraction by solidifying them to a floating solvent drop. This is due to the corresponding melting points which are 18 °C and 13 °C respectively^40^. Aromatic solvents were selected due to their structural similarity to analytes and the possibility of forming π–π interactions which may positively affect extraction efficiency^33^. As expected the highest extraction efficiency was achieved with the use of chlorobenzene and toluene while the area of chromatographic peaks recorded with chlorobenzene extractant was 13–22% higher than in the case of toluene; for chlorobenzene the lowest standard deviations of the recorded peak areas were also recorded. The use of toluene as the extractant requires additional operations to separate it from the extracted matrix. Moving of the extractant to other vessels for this purpose is risky due to the possibility of losing some of the solvent or recontaminating the sample. At the same time all additional operations increase the exposure of the analyst and the environment to the solvent. Taking all the above into accountchlorobenzene was selected as the optimal solvent for extraction.

Optimal solvent volume was selected on the basis of tests performed at 40, 60 and 80 μL. The results of the conducted experiments are presented in Fig. 1b. As expected there was clearly an inverse relationship between the volume of the solvent and the analyte peaks area. Therefore, it was found that the volume of 40 μL is optimal for the conducted experiments. The solubility of chlorobenzene in water is approximately 500 mg/L so the theoretical amount of solvent that can be recovered after extracting a 5 mL water sample is 37.5 μL. After extraction it was possible to collect about 25 μL of chlorobenzene which made it possible to carry out several replications of the analysis using the GC–MS autosampler device. In order to make sure that the determined optimal volume could be used during the wastewater analysisadditional experiments were carried out using influents as well as the wastewater from different treatment stages. For influents it was found necessary to use a solvent volume of 80 μL to obtain 15–20 μL of extract that could be analyzed by GC–MS. It is related to the presence of a rich matrix in wastewater including macromolecular compounds (proteins, fats and carbohydrates), i.e. substances with emulsifying properties which increase the solubility of chlorobenzene^41^. Therefore, it was decided to use a volume of solvent equal to 80 μL in all subsequent experiments. However, when the developed method is used for samples of less polluted water, including surface water, groundwaterand purified sewage it will be possible to use a chlorobenzene volume of 40 μL.

**Figure 2:**
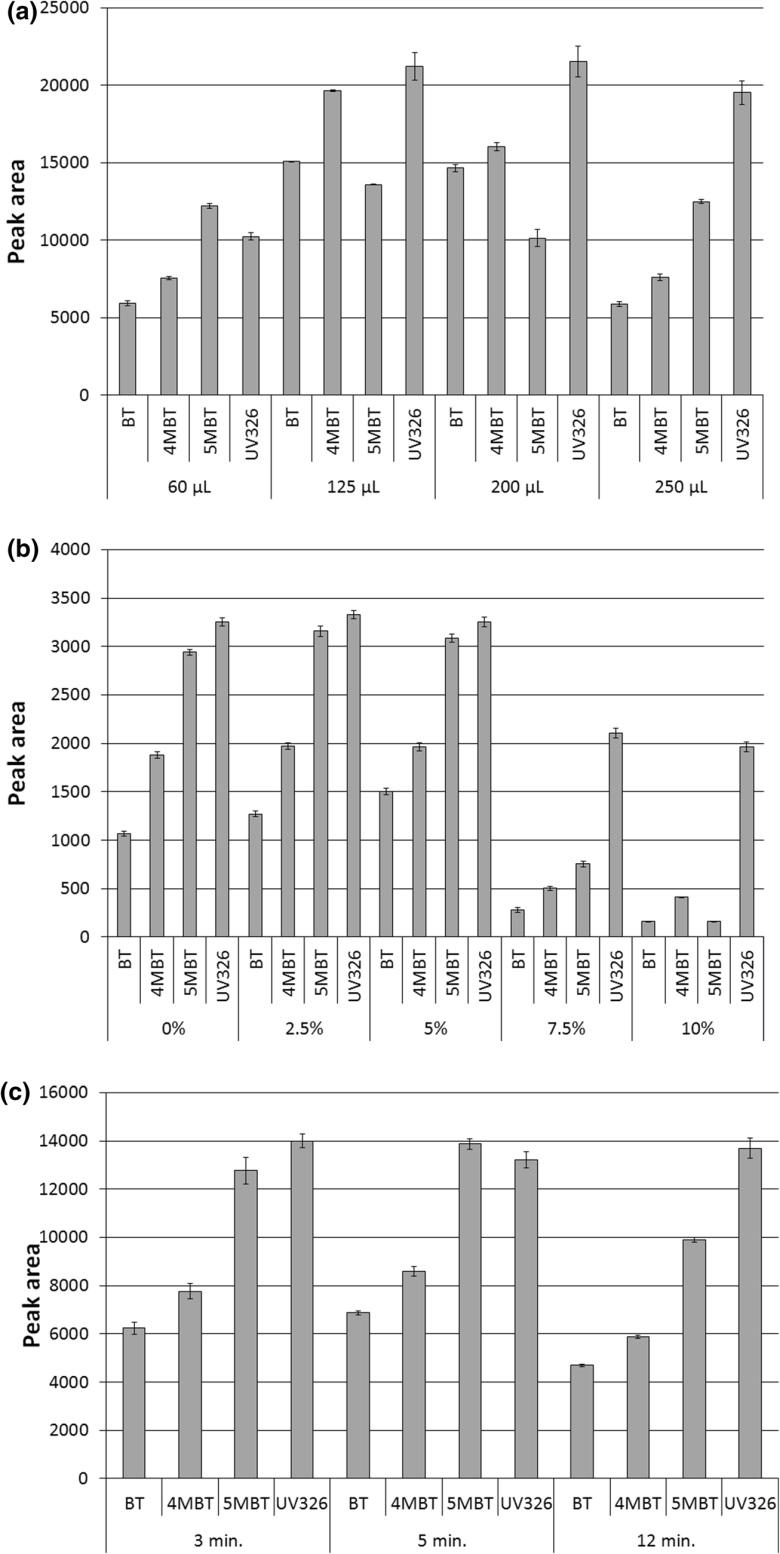
Influence of the acetic anhydride volume (**a**), ionic strength (**b**), and extraction time (**c**) on the efficiency of benzotriazole extraction by the USAEME technique.

The optimization results for acetic anhydride volume, ionic strength and extraction time are shown in Fig. 2. The effect of acetic anhydride volume on the extraction efficiency was investigated in the range from 60 to 250 μL and 125 μL was selected as optimal. It turned out to be sufficient also when the experiments were repeated with samples of raw sewage enriched with analytes at a concentration of 100 μg/L.

Many studies have been carried out in literature on the selection of appropriate salt or buffer solutions for liquid–liquid microextraction^38,42^. As a resultit was found that the optimal choice in this case are phosphoric (V) acid salts with buffering properties. In microextraction methods, especially in USAEME, the use of sodium bicarbonate is disadvantageous because CO_2_ bubbles make it difficult to separate extract from aqueous matrix^43^. However, the influence of ionic strength on the process of benzotriazole microextraction is unclear as available literature provides completely opposite findings^26,33^. In this study the effect of salt addition ranging from 0 to 10% was investigated and the obtained results do not show an unequivocal relationship between the obtained analyte peak areas and salt concentration. Similar results were obtained for concentrations of 0, 2.5 and 5%. In the case of concentrations of 7.5 and 10%, higher peak areas were obtained for BT and 4MBT and lower for 5MBT. In order to definitively settle this issue experiments were repeated for 0 and 10% concentrations with the use of influent wastewater with addition of benzotriazoles. Studies have shown that in the case of a very complex matrix like untreated wastewater, the addition of buffering salt causes precipitation during extraction making it difficult to obtainappropriate volumes of extract. Therefore, in further works no salt was added to the extracted matrix and a similar solution was also selected as optimal when using the DLLME technique^33^.

The relationship between the time of simultaneous extraction,derivatization and extraction efficiency was studied in the range 3–12 min. Extending the simultaneous extraction and derivatization time from 3 to 5 min resulted in an increase in surface areas by about 10%. The surface areas obtained after 12 min process for all tested compounds were about 30% lower than those obtained after 5 min. The decrease in analyte surface areas with sonication time extension indicates that acetyl derivatives of benzotriazoles are likely to partially hydrolyze to free forms upon prolonged contact with aqueous phase. This phenomenon has already been observed in the case of other polar compounds^40^. The 5 min simultaneous extraction and derivatization time was chosen as optimal and used in all subsequent experiments.

### Method validation parameters

Analytical parameters including linearity, precission, LoD, LoQ as well as method recovery were investigated under established optimal conditions. Calibration plots were obtained by spiking the ultrapure water with seven concentration levels between 0.05 and 10 µg/L and performing the extraction and GC–MS analysis. In the case of 5ClBT two chromatographic peaks were recorded. This situation is probably related to the formation of two isomeric acetyl derivatives of 5ClBT, substituted at the 1 and 3 positions of the triazole ring^44^. Calibration plots for this compound were registered and validation parameters were determined based on peak 1, peak 2 and the sum of peaks. The validation data is summarized in Table 1. Calibration plots were linear within the studied concentration ranges with coefficients of determination (r^2^) ≥ 0.9900 for all target compounds.

**Table 1:** USAEME–GC–MS method validation parameters determined with water as sample matrix.

Compound	Linearity		*r*^*2*^	Recovery (%)		CV (%)	LoD (µg/L)	LoQ^a^ (µg/L)
	Slope (SD)^a^	Intercept (SD)^a^		0.2 µg/L	2 µg/L			
BT	402 (6)	12 (14)	0.9983	112 ± 1	106 ± 5	6.4	0.0012	0.05
4MBT	712 (11)	90 (36)	0.9960	108 ± 8	112 ± 2	6.5	0.0007	0.05
5MBT	2616 (42)	− 378 (118)	0.9900	98 ± 8	95 ± 2	8.8	0.0002	0.05
5ClBT (peak1)	281 (4)	17 (38)	0.9971	96 ± 4	102 ± 2	7.7	0.0017	0.05
5ClBT (peak2)	272 (6)	80 (23)	0.9918	116 ± 3	87 ± 7	9.8	0.0018	0.05
5ClBT (∑)	599 (14)	59 (39)	0.9954	108 ± 5	94 ± 6	7.7	0.0009	0.05
UV326	3786 (93)	641 (297)	0.9999	81 ± 6	118 ± 6	4.7	0.0001	0.05
UV329	3608 (87)	1588 (752)	0.9900	106 ± 7	121 ± 6	6.6	0.0001	0.05

*SD* standard deviation, *r*^*2*^ coefficient of determination, *CV* coefficient of variation, *LoD* limit of detection, *LoQ* limit of quantification (the lowest concentration on the calibration plot.

^a^× 10^3^.

The LoD was established as the concentration giving a signal-to-noise ratio (S/N ratio) of 3. It was determined by recording the chromatograms for successive concentrations of analytes until the height of the chromatographic peaks of the specified BTs equalled to three times the height of the noise peaks. The concentrations corresponding to the lowest points of the calibration plots were established as the LoQ values. LoDs values for the tested benzotriazoles ranged from 0.0001 to 0.0018 µg/L. The lowest LoDs were recorded for UV326, UV329 and 5MBT.

The determination precision was established on the basis of CV calculated for developed model (calibration curve). The CV for each point was calculated as the ratio of the root mean squared error to the mean of the concentration calculated on the basis of the model^45^. The CV given in Table 1 was calculated as the average percent value of the variance coefficients determined for seven points of the calibration curves. For the individual compounds tested mean CV values ranging from 4.7 to 9.8% were obtained. As expected the measurements for higher concentrations are characterized by the highest precision. The mean CV value for all tested compounds was 4.7% at 10 µg/L concentration while at 0.05 µg/L it was almost 15%. Recoveries for each compound were determined at two concentration levels: 0.2 and 2 µg/L. They were calculated comparing nominal concentration with the value determined based on calibration curve. The recovery values were between 81 and 116% for the lower concentration and between 87 1nd 118% for the higher value.

### Matrix effect

Municipal and industrial wastewater are matrices with a high degree of pollution and properties strongly differing from those of pure water. A complicated matrix may affect the surface area of the obtained analytical signals as well as the location and course of the baseline. It is assumed that the influence of the matrix at the level of − 20 to 20% is considered acceptable and determinations can be made on the basis of a calibration performed on a simplified matrix. Matrix-matched calibration must be used^46^ with stronger disturbances and tests of the method resistance showed that discrepancies of received signals exceed the determined range whilethe highest exceedances were recorded when influents were the matrix. Therefore, the developed method was validated with the use of six different real matrices (see Supplementary Tables S2 and S3, Supplementary Information). For all municipal wastewater matrix a good linearity was obtained expressed as *r*^2^ value above 0.99. The mean recovery deviation from the value of 100% was the highest for influent wastewater and was approximately 18%. In the case of the remaining matrices it ranged from 8 to 13%, i.e. it did not differ significantly from the values determined for water. The CV values did not exceed 10% which was probably influenced by the higher concentrations within the curve. The average determination sensitivity for the group of target compounds, expressed by the LoD value in the case of municipal wastewater as matrices ranged from 0.006 to 0.014 µg/L. In the case of industrial wastewater as a sample matrix the deviation of validation parameters from those registered for water is higher than in the case of municipal wastewater. In this case values of the recovery were between 77 and 137% for influents and between 89 and 121% for wastewater after flotation. Higher values of CV and LoD were also recorded. It can be seen that the recovery deterioration and sensitivity reduction are quite well correlated with the matrix contamination usedexpressed by the BOD, COD, suspension, nitrogen and phosphorus concentrations (see Supplementary Tables S4, S5, S6, Supplementary Information).

Due to its high polarity and limited volatility liquid chromatography is the most commonly used in BTs analysis and usually combined with MS or MS/MS detection. LC–MS and LC–MS/MS techniques have been gaining increased popularity in recent years due to the high sensitivity and selectivity of determinations. The use of these techniques enables compounds determination without derivatization with high boiling points or thermally unstable compounds. Sample preparation process is simpler when we work with LC techniques compared to GC. The LC–MS/MS methods for the determination of benzotriazoles described in literature are characterized by high sensitivity, good accuracy and precision^19,47^. In the case of LMBTs in municipal wastewater the determined LoDs were between 0.004 and 0.017 µg/L, LoQs in the range 0.011–0.050 µg/L, RSD from 2 to 12%, recovery 50–91%^48^. The BUVs assays were characterized by LoDs values from < 0.001 to 0.002 µg/L, LoQs from 0.001 to 0.007 µg/L, RSD from 1 to 10%, and recovery from 76 to 114%^19^. On the other hand limited availability of LC–MS and LC–MS/MS equipment as well as high cost of determinations and large production of waste solvents are significant obstacles to these techniques. GC–MS systems especially one quadrupole ones are currently common equipment in laboratories conducting environmental determinations. At the same time GC–MS analyzes are cost-friendly, easy to perform and environmentally friendly due to small amount of waste generated. The development of matrix derivatization in recent years facilitated the use of GC in high polarity compound analysis. Table 2 presents a comparison of the developed method with other GC–MS methods using various extraction techniques. Comparing the developed procedure with others described in literature it can be stated that the validation parameters determined in this study are similar or better than in the case of other determinations performed with the GC–MS and GC–MS/MS techniques^22,26,29^. The comparison of isolation techniques shows that SPE, DLLME and USAEME allow similar accuracy, precision and sensitivity determinations while the use of SPME is associated with validation parameters deterioration. However, it should be emphasizedthat procedures based on the use of SPE are multi-stage (conditioning of columns, sample application, elution, concentration of the eluate) and require a large volume of samples (even 2.5 L, on average 1 L) and large volumes of organic solvents (up to 61 mL per one repetition of the procedure)^23^ are used. These facts justify the growing popularity of liquid–liquid microextraction techniques. The DLLME-GC–MS and USAEME-GC–MS procedures have similar analytical characteristics (with a slight advantage of the first technique) while the 20 times higher consumption of organic solvents in the DLLME technique (sum of the extraction and dispersion solvent volumes) should be considered as an advantage of USAEME. A significant improvement of the proposed method compared to the DLLME-based procedure is a simple one-step recovery of the solvent after extraction which is associated with a lower risk of sample contamination and analyte loss^33^.

**Table 2:** Comparison of the proposed method with other approaches based on different extraction techniques and gas chromatography detection.

Analytes	Kind of matrix	Sample volume (mL)	Method	Solvent volume (mL)	*LoDs *(µg/L)	Precission (%)	Relative recovery^a^ (%)	References
BT	Airport run-off	500–1000	SPE–GC–MS^a^	No data	0.1	No data	No data	^39^
LMBTs	River	2500	SPE–GC–MS	8	0.008–0.012	No data	62–70	^35^
LMBTs	Wastewater	200	SPE–GC–MS^b^	25	No data	< 10.0	78–98	^31^
LMBTs	Airport run-off	No data	SPE–GC–MS	61	0.0003–0.01	7.2–12	68–102	^23^
LMBTs	Tap, groundwater, effluents	1000	SPE–GC–MS/MS^c^	19	0.004–0.016	No data	70–122	^22^
LMBTs	Municipal wastewater	1000	SPE–GC–MS/MS	19	< 0.02	1.2–5.1	75–133	^34^
LMBTs	Tap, surface, wastewater	1.6–8	SPME–GC–MS/MS	0	0.2–15	0.1–27.0	57–117	^58^
LMBTs	River, municipal wastewater	500–1000	SPE–GCxGC–ToF–MS	25	0.048–0.112	< 15.7	78–115	^49^
LMBTs	River	200	SPE–GCxGC–ToF–MS	22	0.006–0.038	10.0–12.0	66–102	^6^
LMBTs	Tap, river, municipal wastewater	10	DLLME–GC–MS	1.6	0.007–0.080	< 8.0	92–112	^33^
LMBTs	Municipal and dairy wastewater	5	USAME–GC–MS	0.08	0.006–0.035	< 11.7	77–137	This work
BUVs	Municipal wastewater	1000	SPE–GC–MS/MS	19	0.0015–0.0056	No data	89–110	^22^
BUVs	Municipal wastewater	1000	SPE–GC–MS/MS	19	< 0.0163	0.5–3.8	75–133	^34^
BUVs	Municipal and dairy wastewater	5	USAME–GC–MS	0.08	0.001–0.005	< 9.3	107–124	This work

*LoD* limit of detection.

^a^EI-MS unless otherwise stated.

^b^Ionic liquid stationary phase.

^c^Triple quadrupole.

### Wastewater analysis

**Figure 3:**
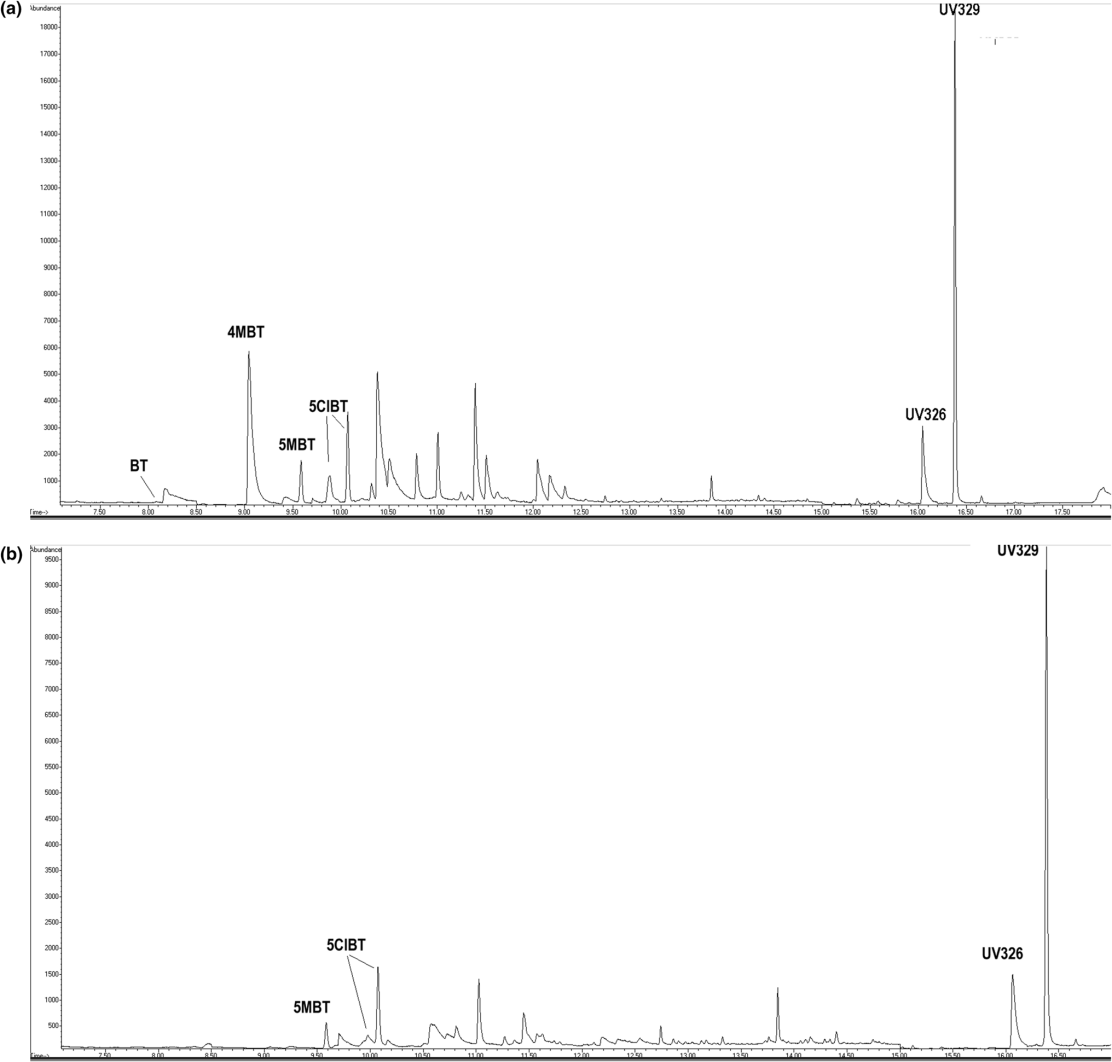
Chromatograms recorded for the influent (**a**) and effluent (**b**) wastewater from WWTP A.

The developed USAEME-GC–MS procedure was used for the simultaneous determination of compounds from the LMBT and BUV groups in wastewater from two municipal wastewater treatment plants and in the outflow of MPP. Figure 3 show chromatograms recorded for the influent and effluent wastewater from WWTP A. Table 3 summarizes the concentrations of the target LMBTs and BUVs in samples of municipal wastewater from WWTP A, WWTP B and in dairy wastewater from MPP. BT, 4MBT and 5MBT determinations were performed on six samples of municipal influents and six samples of effluents (five from WWTP A and one from WWTP B). 5ClBT, UV326, and UV329 determinations were carried out in four samples of each type from WWTP A. Average concentrations of LMBTs in influent wastewater (along with deviations) ranged from 0.055 ± 0.007 µg/L for 5ClBT (N = 2, two values below the LOD) to 2.205 ± 2.335 µg/L for BT. Among the methyl derivatives of BT a higher concentration of 1.117 ± 2.058 µg/L was recorded for 4MBT and for 5MBT it was 0.158 ± 0.136 µg/L. For BUVs the mean concentrations in municipal influents were 0.190 ± 0.082 µg/L and 0.167 ± 0.038 µg/L for UV326 and UV329, respectively (N = 3, one value below LOD). In the treated wastewater the average concentrations of BT (N = 6), 4MBT (N = 6), 5MBT (N = 4) and 5ClBT (N = 2) were 1.065 ± 0.907 µg/L, 0.150 ± 0.171 µg/L, 0.178 ± 0.215 µg/L, and 0.010 µg/L, respectively. The mean concentrations of UV326 (N = 3) and UV329 (N = 2) were 0.040 ± 0.036 µg/L and 0.050 ± 0.028 µg/L, respectively. The LMBTs content in municipal influents determined in this study is similar to the values obtained during research conducted in Spain by Dominguez et al.^31^. Other results that can be found in literature indicate a higher content of these compounds reaching 13 µg/L for BT and over 7 µg/L for other LMBTs^22,47,49^. The content of LMBTs in municipal effluents similar to that recorded in this work was obtained by Liu et al. and Casado et al. for samples from Australia and Spain^22,33,34^. The remaining literature reports give higher concentrations of these compounds in effluents reaching 10 µg/L^47^. The content of BUVs in municipal influents reported in literature is over ten times higher than the values recorded by our study^34^.

**Table 3:** Concentration of benzotriazoles in analyzed municipal and dairy wastewater.

Origin of wastewater samples		Concentration ± standard deviation (µg/L)					
		BT	4MBT	5MBT	5ClBT	UV326	UV329
MWWTP A SC1	Influent	3.46 ± 0.21	0.41 ± 0.06	< LoQ	n.a	n.a	n.a
	Denitrification chamber	3.21 ± 0.34	0.44 ± 0.07	0.7 ± 0.02	n.a	n.a	n.a
	Dephosphatation chamber	3.30 ± 0.31	0.10 ± 0.03	< LoD	n.a	n.a	n.a
	Nitrification chamber	2.28 ± 0.21	0.09 ± 0.05	< LoD	n.a	n.a	n.a
	Effluent	2.17 ± 0.12	0.11 ± 0.02	< LoD	n.a	n.a	n.a
MWWTP A SC2	Influent	2.58 ± 0.26	0.26 ± 0.09	0.12 ± 0.02	< LoD	< LoQ	< LoQ
	Effluent	0.95 ± 0.09	0.02 ± 0.01	0.07 ± 0.02	< LoD	< LoD	< LoD
MWWTP A SC3	Influent	0.26 ± 0.03	0.26 ± 0.02	0.38 ± 0.04	< LoQ	0.12 ± 0.03	0.15 ± 0.04
	Effluent	0.06 ± 0.01	0.06 ± 0.01	0.07 ± 0,.1	< LoQ	0.03 ± 0.01	0.03 ± 0.01
MWWTP A SC4	Influent	0.21 ± 0.03	0.08 ± 0.01	0.05 ± 0.01	< LoQ	0.17 ± 0.04	0.14 ± 0.04
	Effluent	0.41 ± 0.04	0.12 ± 0.03	0.14 ± 0.05	< LoQ	< LoQ	< LoD
MWWTP A SC5	Influent	6.11 ± 0.62	0.38 ± 0.06	0.27 ± 0.04	< LoD	0.28 ± 0.04	0.21 ± 0.04
	Effluent	2.18 ± 0.30	0.10 ± 0.02	< LoD	< LoD	0.08 ± 0.02	0.07 ± 0.02
MWWTP B	Influent	0.61 ± 0.09	5.31 ± 0.84	0.07 ± 0.05	n.a	n.a	n.a
	Retention chamber^a^	1.15 ± 0.18	0.46 ± 0.09	0.64 ± 0.09	n.a	n.a	n.a
	SBR^b^	0.73 ± 0.08	0.48 ± 0.08	0.52 ± 0.09	n.a	n.a	n.a
	Effluent^c^	0.62 ± 0.08	0.49 ± 0.07	0.49 ± 0.06	n.a	n.a	n.a
MPP	Influent	< LoD	0.08 ± 0.02	< LoD	< LoQ	< LoQ	< LoQ
	After flotation	< LoD	< LoD	< LoD	< LoD	< LoD	< LoD

*MWWTP* municipal wastewater treatment plant, *SC* sampling campaign, *SBR* sequencing batch reactor, *n.a.* not analyzed.

^a^After mechanical treatment.

^b^After 190 min of aeration.

^c^From SBR, end of sedimentation.

**Figure 4:**
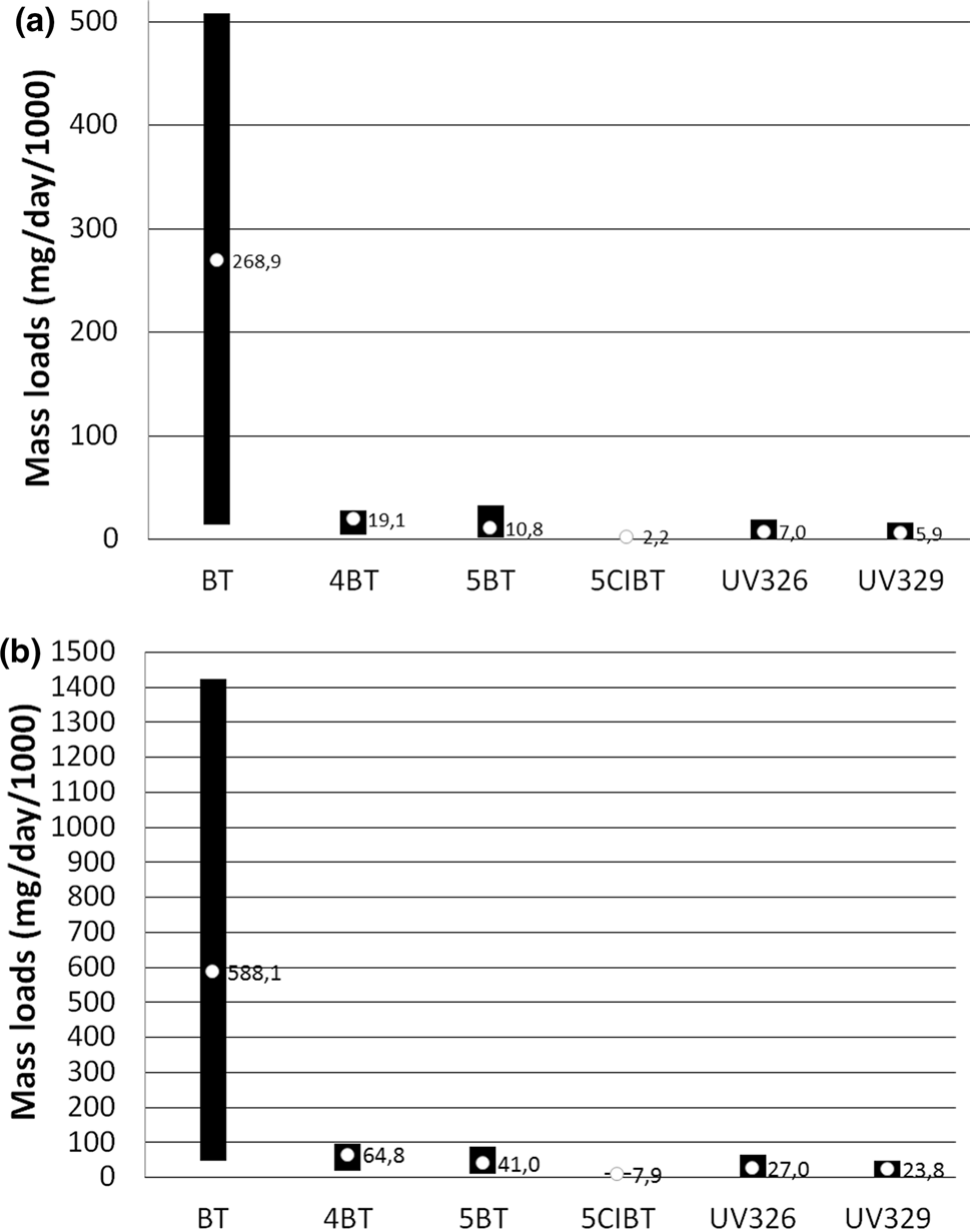
The range and mean mass loads of target compounds flowing with influents into WWTP (**a**) and introduced with effluents into the aquatic environment (**b**).

**Figure 5:**
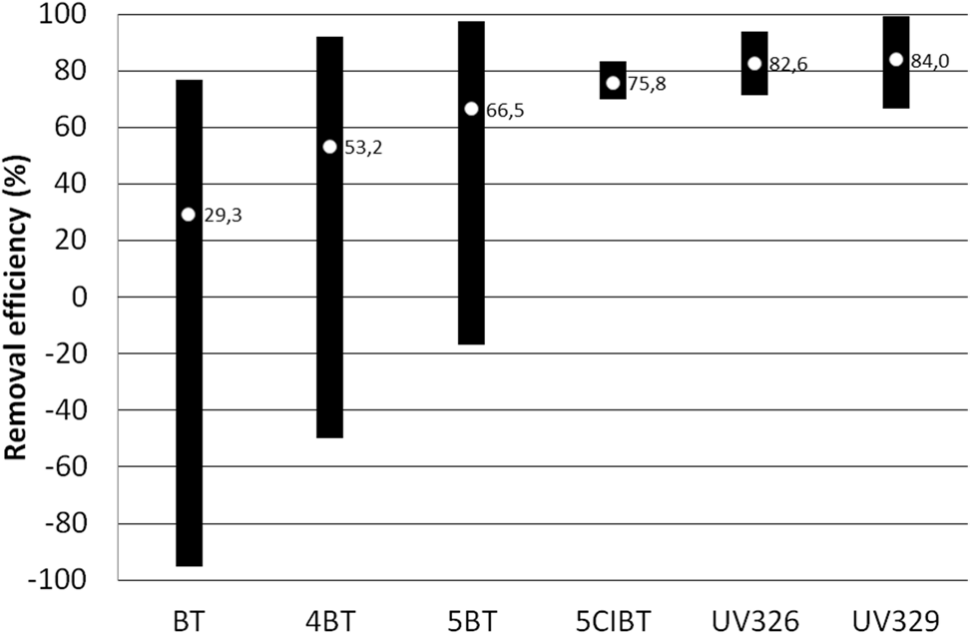
The range and average values of the removal efficiency of benzotriazoles in the WWTP A.

Figure 4a,b show the range and mean ML of target compounds flowing with influents into WWTP A and the range and mean ML of target compounds introduced with effluents from WWTP A into the aquatic environment, respectively. Figure 5 shows the range and average values of removal efficiency (RE) of individual compounds in the WWTP A. When no compound was detected in a specific matrix an appropriate LOD value was inserted into the calculation of ML and RE. The ML values for each target compound were estimated according to Eq. (1). The average ML values calculated for influents ranged from approximately 8 mg/day/1000 inhabitants for 5ClBT to almost 600 mg/day/1000 inhabitants for BT. For the remaining compounds these values were at the level of several dozen mg/day/1000. The lowest mass load introduced into the environment was recorded for 5ClBT and it was 2.2 mg/day/1000 inhabitants. 269 mg/day/1000 inhabitants was was the highest recorded value. The average ML value for the remaining compounds ranged from a few to several mg/day/1000 inhabitants.

Efficiency (RE, %) of the removal of LMBTs and BUVs during the treatment process conducted in WWTP A depends on their tendency to be absorbed by activated sludge as well as biodegradability and physiochemical properties (volatility, polarity, water solubility). RE is defined as negative or low when RE < 20%, moderate when the RE ranged between 20.1% and 70% and high when the RE exceeded 70.1%^50^. As shown in Fig. 5, the average RE value obtained for target compounds allows the removal efficiency of BT, 4MBT and 5MBT to be classified as moderate and 5ClBT, UV326, and UV329 as high. The obtained results correspond with literature data. In laboratory studies of BTs removal conducted by Mazioti et al., BT and ClBT were observed to be significantly removed during aerobic and anoxic activated sludge while 4MBT and 5MBT underwent this process to a small extent^16^. The moderate removal of BT in real conditions observed in this work may result from certain phenomena described later in this section. Biodegradation especially in the aerobic treatment phase is responsible for the removal of BTs in a biological wastewater treatment plant while sorption is of little importance^16^.

In the case of BT and to a lesser extent in the case of 4MBT and 5MBT in some samples higher concentrations were observed in effluents than in influents, i.e. RE values below zero were obtained. “Negative removal” has been previously observed and it could be explained by the varying sorption of the target compounds in activated sludge and their subsequent desorption by successive portions of wastewater^51^. The reason may also be that influents and effluents were collected at the same time while the treatment process in WWTP A takes about 48 h so the effluents came from a completely different portion of influents than taken on a given day^52^. There may also be a situation where complex compounds containing BT in their structure (BUVs, antimicrobial agents, antiparasitic drugs and other) undergo decomposition due to purification processes taking place in WWTPs. This decomposition may result in the formation of BT and its methyl derivatives which increase the concentration of these compounds in effluents. On the basis of the obtained results it can be concluded that removal of BTs by activated sludge processes is not sufficient and it is advisable to use additional technologies for tertiary wastewater treatment.

## Conclusion

This work presents the new USAEME-GC–MS procedure for determination of LMBTs and BUVs in wastewater. The proposed method was optimized and validated using municipal and industrial wastewater at different stages of mechanical–biological treatment. The simple microextraction procedure in combination with in-matrix acylation allows the use of standard GC–MS system with a single quadrupole for determination. The obtained validation parameters make the method a good alternative to more time and costconsuming methods usually used in the determination of benzotriazolesusing the SPE and/or LC–MS/MS techniques. It should be emphasized that the USAEME technique has not so far been used to isolate compounds from the BTs group while BUVs have never been isolated by liquid–liquid microextraction techniques. The developed method was used to determine LMBTs and BUVs in municipal and dairy wastewater from WWTPs located in north-east Poland. All of the target BTs were detected in the tested samples with concentrations similar or lower than those recorded in studies conducted in other countries. An attempt was made to estimate compound loads from the BTs group introduced into the treatment plant with influents and released into the environment with the effluent wastewater together with removal effectiveness. On the basis of the obtained results it can be concluded that the removal of BTs by activated sludge processes is not sufficient and it is advisable to use additional technologies as the tertiary wastewater treatment.

## Methods

### Chemicals and solutions

Analytes: BT, 4MBT, 5MBT on the purity of analytical standards were purchased from Supelco, Germany, and 5ClBT, UV326 and UV329 of 99% purity were obtained from Sigma-Aldrich, Germany. Most solvents: methanol, chloroform, carbon tetrachloride, acetone, chlorobenzene, toluene as well as anhydrous disodium hydrogen phosphate (V) (buffering salt) were purchased from POCH (Poland). Acetic anhydride (V) were provided by Chempur (Poland). Other solvents: 1-undecanol and *n*-hexadecane were obtained from Sigma-Aldrich, Germany. The standards were individually dissolved in methanol to obtain concentrations at 1 mg/mL and prepared stock solutions were stored at − 20 °C for maximum 2 weeks. Working solutions were prepared by diluting stock standard solution in methanol and stored at − 20 °C for maximum 2 weeks. Deionized water was obtained using a purification system (Milli-Q RG, Millipore, USA) andstored in glass bottles. The BTs solutions used in the optimization process and the calibration plots development with the use of milliQ water as a matrix were prepared by adding an appropriate amount of stock standard solution to milliQ water. Solutions for the determination of calibration plots using wastewater as sample matrix were prepared by adding an appropriate amount of stock standard solution to the appropriate wastewater sample. These aqueous solutions were prepared each time immediately before the analyzes were performed.

### Wastewater samples

Average daily municipal wastewater samples were obtained from municipal WWTPs located in two cities in north-east Poland. Treatment processes used by WWTPs include mechanical purification (such as grates, sieves, sand traps, settling tanks and grease separators) as well as biological purification through the use of activated sludge with no tertiary treatment. After the completion of treatment processes effluents are discharged into local rivers belonging to the catchment area of the Vistula River which is a part of the Baltic Sea drainage basin. WWTP A is located in a city with the population of 300,000 and its daily capacity equals 100,000 m^3^/day. Its average processing is approximately 70.000 m^3^/day. The work of the plant is based on active sludge technology (flow system) assuming the load of BOD_5_ equal to 30.000 kg O_2_/day, suspension load of 55,000 kg/day, total Kjeldahl nitrogen 6000 kg/day and total phosphorus 850 kg/day. Treatment efficiency meets the effluent standards required by Polish legislation for a plant of this size. Average parameters of raw wastewater are: COD 690.0 mgO_2_/L, pH 6.8, total suspended solids (TSS) 460.0 mg/L, total nitrogen (TN) 89.5 mg/L, total phosphorus (TP) 11.9 mg/L^53^. WWTP B is also located in north-east Poland and purifies wastewater from 21.000 inhabitants andprocesses approximately 5000 m^3^ daily. Theoretical capacity of WWTP B equals 6600 m^3^/day. The main source of contamination affecting the drains is municipal and industrial (dairy, charcoal and briquette production, bio-fuels). Treatment efficiency meets the effluent standards required by Polish legislation for a plant of this size. Treatment processes used by WWTPs include mechanical purification as well as biological purification through the use of activated sludge in sequential batch reactors (SBR). Average parameters of raw wastewater are: COD 1079 mgO_2_/L, pH 6.9, TSS 430.0 mg/L, TN 71.0 mg/L, and TP 11.8 mg/L^54^. The presented Milk Processing Plant (MPP) is one of the twenty largest dairiess in Europe^55^ and processes 54 million liters of milk per year. The MPP discharges pretreated dairy wastewater to a municipal WWTP and the average amount is 430 m^3^/day which is about 13% of the total volume of wastewater flowing into the given WWTP. In terms of BOD_5_ and COD load dairy sewage has 7% share in the pollution load^56^. Wastewater samples were gathered using glass samplers, placed into glass bottles and then transported to the laboratory. Obtained samples were filtered through a 0.45 µm pore membrane filter and fixed with concentrated HCl to pH = 2. Samples were then stored in a freezer at − 20 °C. pH and electrolytic conductivity (EC) were measured using probe of Hach HQd Meter and IntelliCAL Smartprobes type, Germany. Analyses of BOD_5_; COD; orthophosphates; TP; ammonia nitrogen; nitrates; TN; TSS were carried out in accordance with APHA^57^.

### Procedure of benzotriazole microextraction with in situ derivatization

For the simultaneous extraction and derivatization of BT, 4MBT, 5MBT, 5ClBT, UV326, and UV329 an aliquots of 5 mL of examined liquid sample was placed in 10-mL glass centrifuge test-tube. The extraction solvent (chlorobenzene, 80 µL) and the derivatization reagent (acetic anhydride, 125 µL) were added to such prepared samples and mixed. Nextthe tubes were immersed in an ultrasonic bath (Polsonic, Sonic-3, Poland). Extractions were performed at 42 kHz of ultrasound frequency and 230 W power for the duration of 5 min at room temperature. Emulsions were disrupted by centrifugation at 6000 rpm for 5 min in an MPW-250 Med. Instruments (Poland) laboratory centrifuge. The organic phase was settled at the bottom of the conical tube and the organic solvent along with the extracted analytes were removed using a 100 µL Hamilton syringe (USA) and transferred into chromatographic vial equipped with an insert with a capacity of 150 µL.

### GC–MS conditions

Analysis was performed with a HP 6890 gas chromatograph with a mass spectrometric detector MSD5973 and HP 7673 autosampler (Agilent Technologies, USA). This device was equipped with HP-5MS column (5% phenylmethylsiloxane) with dimensions 30 m length × 0.25 mm with 0.25 µm film thickness and split/splitless injector. The injector worked in splitless mode, injection volume was 1 µL. Purity 99.999% helium was used as carrier gas at flow rate 1 mL/min,the injector temperature was 250 °C, the oven temperature was programmed at 80 °C, increased by 10 °C/min to 180 °C and 20 °C/min to 280 °C. The total run time was 17.00 min and the retention times were as follows: 8.00 min (BT), 9.16 min (4MBT), 9.46 min (5MBT), 9.85 min and 9.91 min (5ClBT), 15.99 min (UV326), 16.46 min (UV329). The electron impact source temperature was 230 °C with electron energy of 70 eV. The quadrupole temperature was 150 °C, and the GC interface temperature was 280 °C. The MS detector worked in Selected Ion Monitoring (SIM) mode. The SIM chromatogram of the mili-Q water spiked with mixture of target compounds (100 μg/L each) is presented in Supplementary Fig. S1 (Supplementary Information). Registered EI-MS spectra of analytes are presented in Supplementary Fig. S2 (Supplementary Information). All signals were well separated and single, good shape, and repeatable peaks for acetyl derivatives of BT, 4MBT, 5MBT, UV329 were obtained. UV326 did not undergo an acylation reaction due to a steric hindrance that prevented the derivatization reagent from accessing the OH group (see Supplementary Fig. S2, Supplementary Information). The chromatographic and mass spectrometric parameters together with some charasteristics and structures of target compounds are included in Supplementary Table S7 (Supplementary Information).

### Calculation of mass loads

Mass loads (*ML*) were calculated by multiplying concentrations of each target compound $i$ found in wastewater samples (*C*, µg/L) the average daily flow rate (*Q*, m^3^/day) of the tasted plant and the population served by the WWTP (*P*). *ML* values were normalized using the community equivalent (1000) according to Eq. (1) and they were expressed in mg/day/1000 inhabitants.

1 $$ML_{i} = \frac{{C_{i} \times Q \times 1000}}{P}.$$

The *ML* values of BTs flowing into WWTP A along with influents and the *ML* values of BTs introduced into the environment along with treated wastewater were determined.

### Quality assurance

Apparatus (GC–MS) and procedural blanks were registered before analysis of samples or standards and several times during analysis cycle to check no carry-over effect and purity of reagents and equipment used. Brand new test tubes and only glass materials were used in experiments to avoid possible bacground contamination and losses of analytes by sorption on plastic. Concentrations of studied compounds in all influent and effluent samples they were calculated on the basis of the calibration plot registered in analyzed matrix. The determinations in the wastewater collected from the nitrification chamber and from SBR were performed based on plots recorded for effluents. Determinations in wastewater collected from retention chamber (after mechanical tratment) were done on the basis of influents calibration.

## Supplementary Information

Supplementary Information.

## References

[1] Żyłka R, Dąbrowski W, Karolinczak B (2020). Modeling of electric energy consumption during dairy wastewater treatment plant operation. Energies.

[2] Asimakopoulos AG, Wang L, Thomaidis NS, Kannan K (2013). Benzotriazoles and benzothiazoles in human urine from several countries: A perspective on occurrence, biotransformation, and human exposure. Environ. Int..

[3] Santiago MR (2018). Treatment of dairy wastewater by oxygen injection: Occurrence and removal efficiency of a benzotriazole based anticorrosive. Water.

[4] Shi Z-Q, Liu Y-S, Xiong Q, Cai W-W, Ying G-G (2019). Occurrence, toxicity and transformation of six typical benzotriazoles in the environment. A review. Sci. Total Environ..

[5] Briguglio I (2015). Benzotriazole: An overview on its versatile biological behavior. Eur. J. Med. Chem..

[6] Matamoros V, Jover E, Bayona JM (2010). Occurrence and fate of benzothiazoles and benzotriazoles in constructed wetlands. Water Sci. Technol..

[7] Reemtsma TH, Miehe U, Duennbier U, Jekel M (2010). Polar pollutants in municipal wastewater and the water cycle: Occurrence and removal of benzotriazoles. Water Res..

[8] Cantwell MG, Sullivan JC, Burgess RM (2015). Benzotriazoles: History, environmental distribution, and potential ecological effects. Compr. Anal. Chem..

[9] Montesdeoca-Esponda S, del Toro-Moreno A, Sosa-Ferrera Z, Santana-Rodríguez JJ (2013). Development of a sensitive determination method for benzotriazole UV stabilizers in environmental water samples with stir bar sorption extraction and liquid desorption prior to ultra-high performance liquid chromatography with tandem mass spectrometry. J. Sep. Sci..

[10] Lai H-J (2014). Occurrence and dissipation of benzotriazoles and benzotriazoli ultraviolet stabilizers in biosolid-amended soils. Environ. Toxicol. Chem..

[11] Rhodes-Dicker L, Passeport E (2019). Effects of cold-climate environmental factors temperature and salinity on benzotriazole adsorption and desorption in bioretention cells. Ecol. Eng..

[12] Lempart, A., Kudlek, E. & Dudziak, M. Determination of micropollutants in solid and liquid samples from swimming pool systems. In *Proc. of the 2nd Int. Elect. Conf. Water Sci.* 16–30; *Sciforum Electronic Conference Series*, Vol. 2 (2017).

[13] Corsi SR, Harwell GR, Geis SW, Bergman D (2006). Impacts of aircraft deicer and anti-icer runoff on receiving waters from Dallas/Fort Worth International Airport, Texas, USA. Environ. Toxicol. Chem..

[14] Suma BV, Natesh NN, Madhavan V (2011). Benzotriazole in medicinal chemistry: An overview. J. Chem. Pharm. Res..

[15] Roshani B, McMasterb I, Rezaeib E, Soltan J (2014). Catalytic ozonation of benzotriazole over alumina supported transition metal oxide catalysts in water. Sep. Purif. Technol..

[16] Mazioti A, Stasinakis A, Gatidou G, Thomaidis N, Andersen H (2015). Sorption and biodegradation of selected benzotriazoles and hydroxybenzothiazole in activated sludge and estimation of their fate during wastewater treatment. Chemosphere.

[17] Tangtian H, Liang B, Liu W, Shin P, Wu R (2012). Estrogenic potential of benzotriazole on marine medaka (*Oryzias melastigma*). Ecotoxicol. Environ. Saf..

[18] Fent K, Chew G, Li J, Gomez E (2014). Benzotriazole UV-stabilizers and benzotriazole: Antiandrogenic activity in vitro and activation of aryl hydrocarbon receptor pathway in zebrafish eleuthero-embryos. Sci. Total Environ..

[19] Liu R (2014). Determination of nine benzotriazole UV stabilizers in environmental water samples by automated on-line solid phase extraction coupled with high-performance liquid chromatography–tandem mass spectrometry. Talanta.

[20] Molins-Delgado D, Távora J, Díaz-Cruz MS, Barceló D (2017). UV filters and benzotriazoles in urban aquatic ecosystems: The footprint of daily use products. Sci. Total Environ..

[21] Giger, W. *Eawag Research* (Swiss Federal Institute of Aquatic Science and Technology, 2006).

[22] Liu Y-S, Ying G-G, Shareef A, Kookana RS (2011). Simultaneous determination of benzotriazoles and ultraviolet filters in ground water, effluent and biosolid samples using gas chromatography–tandem mass spectrometry. J. Chromatogr. A.

[23] Sulej AM, Polkowska Ż, Astel A, Namieśnik J (2013). Analytical procedures for the determination of fuel combustion products, anti-corrosive compounds, and de-icing compounds in airport runoff water samples. Talanta.

[24] Ababneh AN, Abu-Dalo MA, Horn C, Hernandez MT (2019). Polarographic determination of benzotriazoles and their sorption behavior on granular activated carbon. Int. J. Environ. Sci. Technol..

[25] Esmaile N, Shabaneh S, Mofavvaz S, Sohrabi MR, Torabi B (2020). Spectrophotometric determination of trace amounts of benzotriazole in aqueous solutions using gold nanoparticles: Artificial neural network modeling. ChemistrySelect.

[26] Lu J, Wang M-M, Wang Q, Li H-P, Yang Z-G (2018). Determination of benzotriazole and its derivatives in aqueous sample with air-assisted liquid–liquid microextraction followed by high-performance liquid chromatography. Chin. J. Anal. Chem..

[27] Lu Z (2019). Occurrence of substituted diphenylamine antioxidants and benzotriazole UV stabilizers in Arctic seabirds and seals. Sci. Total Environ..

[28] Gatidou G, Anastopoulou P, Aloupi M, Stasinakis AS (2019). Growth inhibition and fate of benzotriazoles in *Chlorella sorokiniana* cultures. Sci. Total Environ..

[29] Ahmad SM, Calado BB, Oliveira MN, Neng NR, Nogueira J (2020). Bar adsorptive microextraction coated with carbon-based phase mixtures for performance-enhancement to monitor selected benzotriazoles, benzothiazoles, and benzenesulfonamides in environmental water matrices. Molecules.

[30] Pacheco-Juárez J, Montesdeoca-Esponda M, Torres-Padrón ME, Sosa-Ferrera Z, Santana-Rodríguez JJ (2019). Analysis and occurrence of benzotriazole ultraviolet stabilisers in different species of seaweed. Chemosphere.

[31] Domínguez C, Reyes-Contreras C, Bayona JM (2012). Determination of benzothiazoles and benzotriazoles by using ionic liquid stationary phases in gas chromatography mass spectrometry. Application to their characteriza-tion in wastewaters. J. Chromatogr. A.

[32] Matamoros V, Jover E, Bayona JM (2010). Part-per-trillion determination of pharmaceuticals, pesticides, and related organic contaminants in river water by solid-phase extraction followed by comprehensive two-dimensional gas chromatography time-of-flight mass spectrometry. Anal. Chem..

[33] Casado J (2014). Determination of benzotriazoles in water samples by concurrent derivatization-dispersive liquid–liquid microextraction followed by gas chromatography–mass spectrometry. J. Chromatogr. A.

[34] Liu Y-S, Ying G-G, Shareef A, Kookana RS (2012). Occurrence and removal of benzotriazoles and ultraviolet filters in a municipal wastewater treatment plant. Environ. Pollut..

[35] Kiss A, Fries E (2009). Occurrence of benzotriazoles in the rivers Main, Hengstbach, and Hegbach (Germany). Environ. Sci. Pollut. Res. Int..

[36] Carasek E, Bernardi G, Morelli D, Merib J (2021). Sustainable green solvents for microextraction techniques: Recent developments and applications. J. Chromatogr. A.

[37] Pacheco-Fernández I, González-Martín R, de Silva FA, Freire MG, Pino V (2021). Insights into coacervative and dispersive liquid-phase microextraction strategies with hydrophilic media—A review. Anal. Chim. Acta.

[38] Regueiro J, Llompart M, Garcia-Jares C, Monteagudo JC, Cela R (2008). Ultrasound-assisted emulsification–microextraction of emergent contaminants and pesticides in environmental waters. J. Chromatogr. A.

[39] Breedveld GD, Roseth R, Sparrevik M, Hartnik T, Hem LJ (2003). Persistence of the de-icing additive benzotriazole at an abandoned airport. Water Air Soil Pollut..

[40] Kotowska U, Kapelewska J, Kotowski A, Pietuszewska E (2019). Rapid and sensitive analysis of hormones and other emerging contaminants in groundwater using ultrasound-assisted emulsification microextraction with solidification of floating organic droplet followed by GC–MS detection. Water.

[41] Kotowska U, Biegańska K, Isidorov VA (2012). Screening of trace organic compounds in municipal wastewater by gas chromatography–mass spectrometry. Pol. J. Environ. Stud..

[42] Kotowska U, Kapelewska J, Sturgulewska J (2014). Determination of phenols and pharmaceuticals in municipal wastewaters from Polish treatment plants by ultrasound-assisted emulsification–microextraction followed by GC–MS. Environ. Sci. Pollut. Res..

[43] Spietelun A, Marcinkowski Ł, de la Guardia M, Namieśnik J (2014). Green aspects, developments and perspectives of liquid phase microextraction techniques. Talanta.

[44] Benson FR, Hartzel LW, Savell WL (1952). 5,6-Dimethylbenzotriazole and its acyl derivatives. J. Am. Chem. Soc..

[45] UCLA Institute for Digital Research & Education, Statistical Consulting. https://stats.idre.ucla.edu. Accessed 22 Jan 2021 (2020).

[46] Woźniak MK (2020). Development and validation of a GC–MS/MS method for the determination of 11 amphetamines and 34 synthetic cathinones in whole blood. Forensic Toxicol..

[47] Carpinteiro I, Abuin B, Ramil M, Rodríguez I, Cela R (2012). Simultaneous determination of benzotriazole and benzothiazole derivatives in aqueous matrices by mixed-mode solid-phase extraction followed by liquid chromatography–tandem mass spectrometry. Anal. Bioanal. Chem..

[48] Weiss S, Jakobs J, Reemtsma T (2006). Discharge of three benzotriazole corrosion inhibitors with municipal wastewater and improvements by membrane bioreactor treatment and ozonation. Environ. Sci. Technol..

[49] Jover E, Matamoros V, Bayona J (2009). Characterization of benzothiazoles, benzotriazoles and benzosulfonamides in aqueous matrixes by solid-phase extraction followed by comprehensive two-dimensional gas chromatography coupled to time-of-flight mass spectrometry. J. Chromatogr. A.

[50] Papageorgiou M, Kosma C, Lambropoulou D (2016). Seasonal occurrence, removal, mass loading and environmental risk assessment of 55 pharmaceuticals and personal care products in a municipal wastewater treatment plant in Central Greece. Sci. Total Environ..

[51] Wu Q, Lam JCW, Kwok KY, Tsui MMP, Lam PKS (2017). Occurrence and fate of endogenous steroid hormones, alkylphenol ethoxylates, bisphenol A and phthalates in municipal sewage treatment systems. J. Environ. Sci..

[52] Kotowska U, Kapelewska J, Sawczuk R (2020). Ocurrence, removal, and environmental risk of phthalates in wastewaters, landfill leachates, and groundwater in Poland. Environ. Pollut..

[53] Struk-Sokołowska J (2020). Impact of differences in speciation of organic compounds in wastewater from large WWTPs on technological parameters, economic efficiency and modelling of chemically assisted primary sedimentation process. J. Environ. Chem. Eng..

[54] Struk-Sokołowska J, Mielcarek A, Wiater J, Rodziewicz J (2018). Impacts of dairy wastewater and pre-aeration on the performance of SBR. Desalin. Water Treat..

[55] Struk-Sokołowska J (2011). Changes of COD fractions share during municipal wastewater treatment with big dairy wastewater participation. Rocznik Ochrona Środowiska.

[56] Struk-Sokołowska J, Rodziewicz J, Mielcarek A (2018). Effect of dairy wastewater on changes in COD fractions in technical-scale SBR type reactors. Water Sci. Technol..

[57] Rice, E. W., Baird, R. B., Eaton, A. D. & Clesceri, L. S. *Standard Methods for the Examination of Water and Wastewater.* 23nd Edn. (Ame. Pub. Hea. Ass. (APHA), Ame. Wat. Wor. Ass. (AWWA), Wat. Envir. Fed. (WEF), 2017).

[58] Naccarato A, Gionfriddo E, Sindona G, Tagarelli A (2014). Simultaneous determination of benzothiazoles, benzotriazoles and benzosulfonamides by solid phase microextraction-gas chromatography-triple quadrupole mass spectrometry in environmental aqueous matrices and human urine. J. Chromatogr. A.

