# Supplementary material for: Simultaneous determination of low molecule benzotriazoles and benzotriazole UV stabilizers in wastewater by ultrasound-assisted emulsification microextraction followed by GC–MS detection
Source: Sci Rep. 2021 May 12;11:10098. doi: 10.1038/s41598-021-89529-1 (PMC8114919; doi:10.1038/s41598-021-89529-1)
Supplement: Supplementary file 1 — Supplementary Information. [file 41598_2021_89529_MOESM1_ESM.docx]

**Simultaneous determination of low molecule benzotriazoles and benzotriazole UV stabilizers in wastewater
by ultrasound-assisted emulsification microextraction followed by GC-MS detection**

Kotowska Urszula^1^*, Struk-Sokołowska Joanna^2^, Piekutin Janina^2^

^1^ *University of Bialystok, Faculty of Chemistry, Department of Analytical and Inorganic Chemistry, Ciołkowskiego 1K Street, 15-245 Bialystok, Poland;*

*^2^ Bialystok University of Technology, Faculty of Civil Engineering and Environmental Sciences, Department of Environmental Engineering Technology, Wiejska 45E, 15-351 Bialystok, Poland*

*Corresponding author, e-mail: ukrajew@uwb.edu.pl; tel.: +48 85 738 81 11

**SUPPLEMENTARY MATERIAL**

Figures: S1, S2

Tables: S1, S2, S3, S4, S5, S6, S7


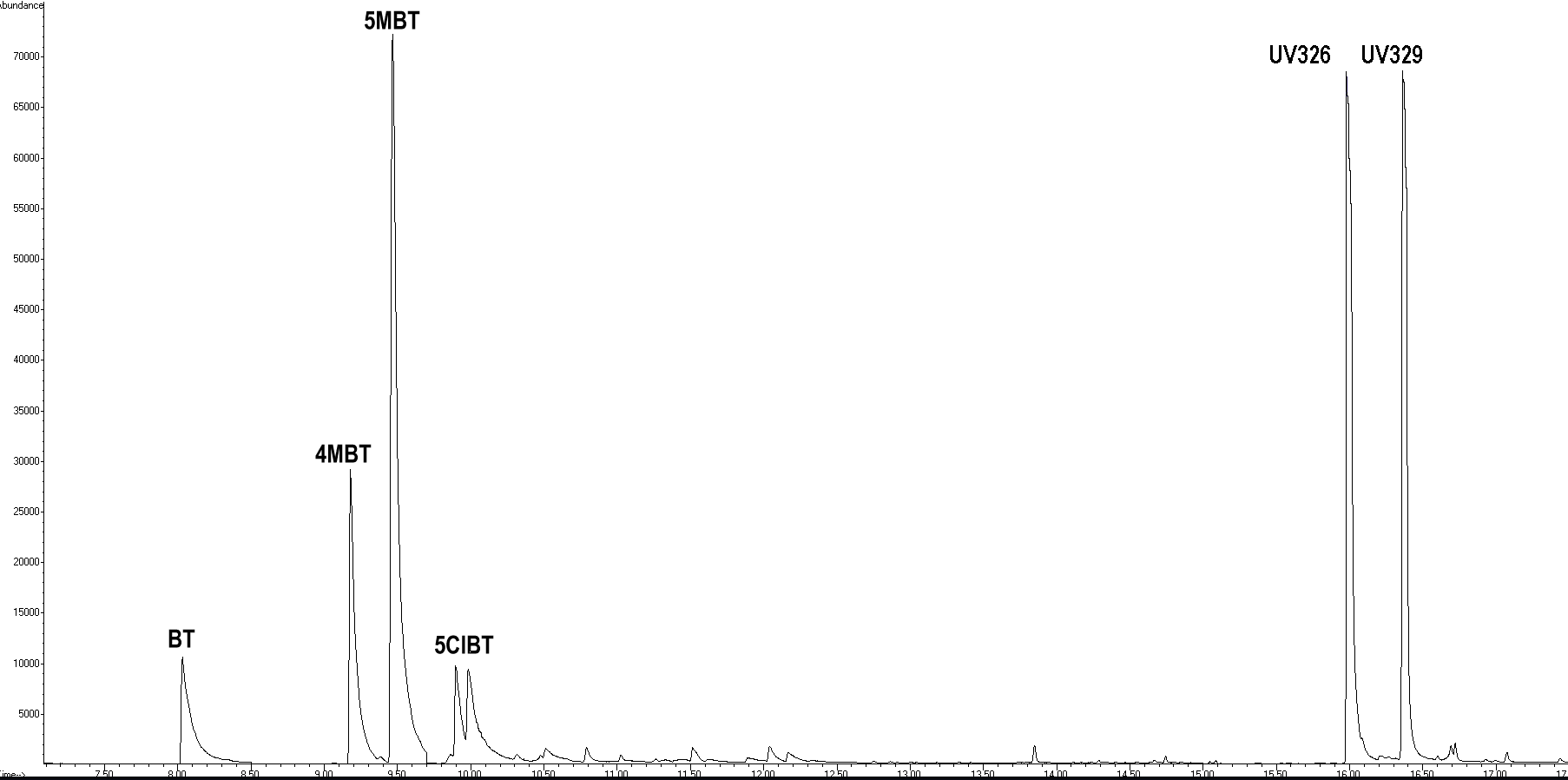


Figure S1. The chromatogram of the mili-Q water spiked with mixture of target compounds registered in Selected Ion Monitoring (SIM) mode


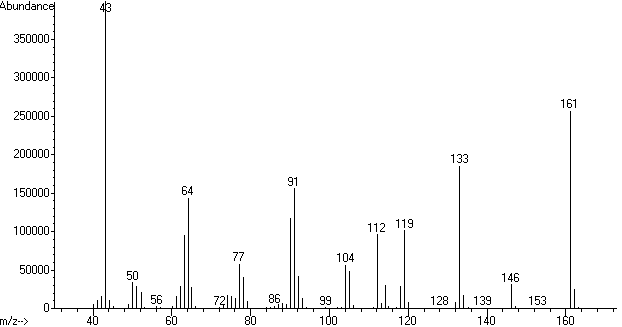

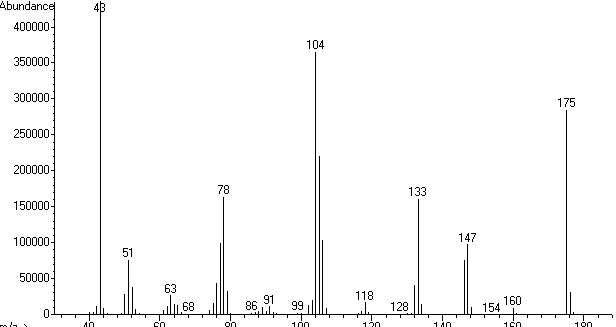

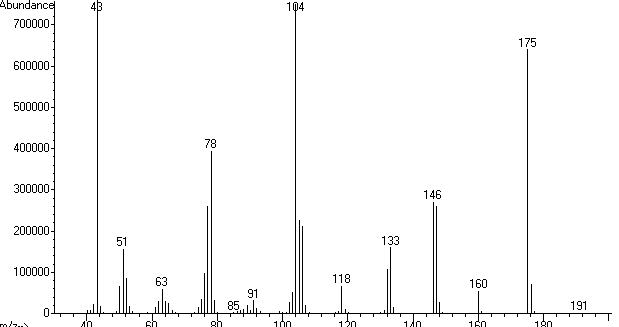


**C**

**B**

**A**


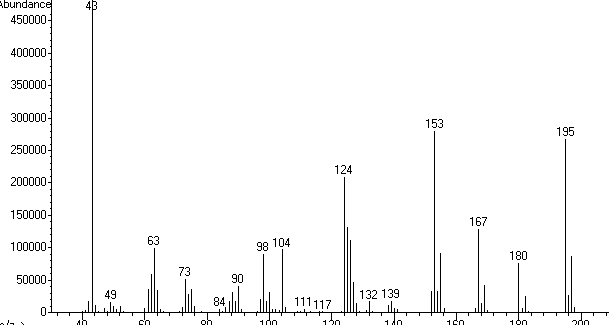

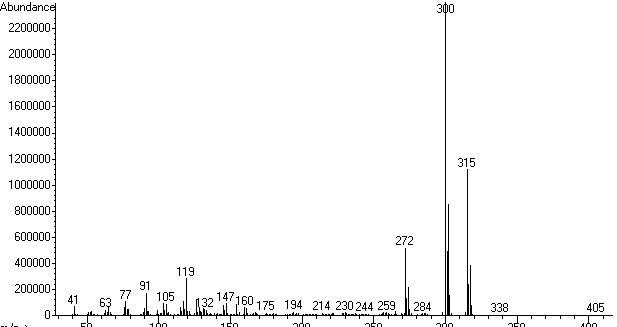

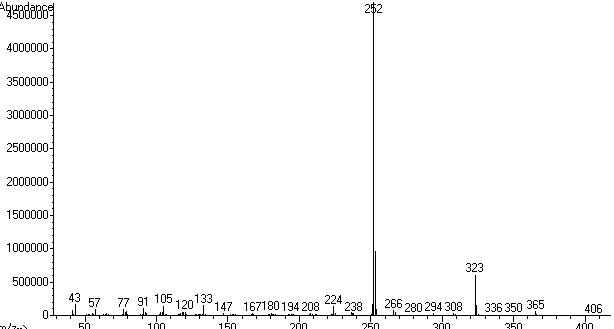


**F**

**E**

**D**

Figure S2. EI-MS spectra of benzotriazole (A), 4-methylbenzotriazole (B), 5-methylbenzotriazole (C), 5-chlorobenzotriazole (D), UV326 (E), and UV329 (F)

Table S1. Properties of solvents tested for benzotriazoles extraction by USAEME technique

| **Solvent** | **CAS** | **Molar mass (g/mol)** | **Melting point (^o^C)** | **Boiling point (^o^C)** | **Density (g/mL)** | **Solubility in water in 25°C (mg/L)** |
| --- | --- | --- | --- | --- | --- | --- |
| Carbon tetrachloride | 56-23-5 | 153.80 | -22.2 | 76.8 | 1.594 | 793 |
| Chlorobenzene | 108-90-7 | 112.55 | -45.2 | 131.6 | 1.110 | 498 |
| Chloroform | 67-66-3 | 119.37 | -63.5 | 61.1 | 1.479 | 795 |
| *n*-Hexadecane | 544-76-3 | 226.41 | 18.0 | 286.8 | 0.773 | 2·10^-5^ |
| Toluene | 108-88-3 | 92.14 | -94.9 | 110.6 | 0.867 | 526 |
| 1-Undecanol | 112-42-5 | 172.31 | 13.0 | 243.0 | 0.830 | 19.0 |

Table S2. USAEME-GC-MS method validation parameters determined with the municipal wastewater as sample matrix

| **Compound** |  | **Linear range (µg/L)** | ***r^2^*** | **Recovery* (%)** | ***CV* (%)** | ***LoD* (µg/L)** |  | **Linear range (µg/L)** | ***r^2^*** | **Recovery* (%)** | ***CV* (%)** | ***LoD* (µg/L)** |
| --- | --- | --- | --- | --- | --- | --- | --- | --- | --- | --- | --- | --- |
|  |  |  |  |  |  |  |  |  |  |  |  |  |
|  |  | Influent | | | | |  | Denitrification chamber | | | | |
| BT |  | 0.1-100 | 0.9902 | 121±11 | 10.1 | 0.015 |  | 0.1-100 | 0.9909 | 107±5 | 8.4 | 0.019 |
| 4MBT |  | 0.1-100 | 0.9911 | 120±9 | 8.6 | 0.020 |  | 0.1-100 | 0.9931 | 116±2 | 6.8 | 0.019 |
| 5MBT |  | 0.1-100 | 0.9892 | 82±8 | 9.1 | 0.011 |  | 0.1-100 | 0.9932 | 92±9 | 9.9 | 0.007 |
| 5ClBT |  | 0.5-100 | 0.9917 | 89±9 | 9.9 | 0.033 |  | 0.5-100 | 0.9919 | 84±6 | 6.7 | 0.024 |
| UV326 |  | 0.1-100 | 0.9984 | 118±6 | 9.3 | 0.005 |  | 0.1-100 | 0.9986 | 120±7 | 6.3 | 0.002 |
| UV329 |  | 0.1-100 | 0.9906 | 110±8 | 8.1 | 0.002 |  | 0.1-100 | 0.9926 | 112±6 | 9.2 | 0.002 |
|  |  | Dephosphatation chamber | | | | |  | Effluent | | | | |
| BT |  | 0.05-100 | 0.9899 | 111±10 | 7.6 | 0.009 |  | 0.05-100 | 0.9929 | 112±8 | 6.8 | 0.010 |
| 4MBT |  | 0.05-100 | 0.9943 | 106±6 | 7.0 | 0.011 |  | 0.05-100 | 0.9951 | 106±2 | 7.5 | 0.010 |
| 5MBT |  | 0.05-100 | 0.9899 | 97±8 | 9.0 | 0.008 |  | 0.05-100 | 0.9920 | 102±7 | 4.9 | 0.006 |
| 5ClBT |  | 0.05-100 | 0.9965 | 81±8 | 4.7 | 0.015 |  | 0.05-100 | 0.9987 | 103±6 | 9.1 | 0.009 |
| UV326 |  | 0.05-100 | 0.9990 | 107±8 | 9.0 | 0.002 |  | 0.05-100 | 0.9985 | 111±7 | 9.3 | 0.001 |
| UV329 |  | 0.05-100 | 0.9927 | 109±8 | 8.2 | 0.001 |  | 0.05-100 | 0.9958 | 112±5 | 6.8 | 0.001 |

*r^2^* –coefficient of determination; *CV* - coefficient of variation*; LoD* – limit of detection.

*C = 2 µg/L

Table S3. USAEME-GC-MS method validation parameters determined for industrial (diary) wastewater as sample matrix

| **Compound** | **Linear range**  **(µg/L)** | ***r^2^*** | **Recovery* (%)** | ***CV* (%)** | ***LoD* (µg/L)** |  | **Linear range (µg/L)** | ***r^2^*** | **Recovery* (%)** | ***CV* (%)** | ***LoD* (µg/L)** |
| --- | --- | --- | --- | --- | --- | --- | --- | --- | --- | --- | --- |
| Influent | | | | | |  | After flotation | | | | |
| BT | 0.1-100 | 0.9907 | 137±11 | 8.4 | 0.030 |  | 0.1-100 | 0.9912 | 110±9 | 7.4 | 0.009 |
| 4MBT | 0.1-100 | 0.9835 | 126±12 | 9.9 | 0.020 |  | 0.1-100 | 0.9859 | 111±10 | 7.8 | 0.021 |
| 5MBT | 0.1-100 | 0.9900 | 77±9 | 10.5 | 0.009 |  | 0.1-100 | 0.9912 | 99±11 | 9.9 | 0.008 |
| 5ClBT | 0.5-100 | 0.9911 | 84±6 | 11.7 | 0.035 |  | 0.5-100 | 0.9909 | 89±8 | 9.7 | 0.025 |
| UV326 | 0.01-100 | 0.9921 | 128±7 | 9.7 | 0.003 |  | 0.01-100 | 0.9991 | 119±8 | 9.3 | 0.002 |
| UV329 | 0.01-100 | 0.9906 | 119±8 | 7.5 | 0.003 |  | 0.01-100 | 0.9944 | 121±8 | 8.2 | 0.002 |

*r^2^* –coefficient of determination; *CV* - coefficient of variation; *LoD* – limit of detection.

* C = 2 µg/L

Table S4. Average physicochemical indicators of municipal wastewater treated in WWTP A (PE>100.000) with activated sludge (flow technology) used in the study

| Parameter | Wastewater | | | | |
| --- | --- | --- | --- | --- | --- |
|  | influent | collected from denitrification chamber | collected from dephosphatation chamber | collected from nitrification chamber | effluent |
| pH | 7.46 | 8.16 | 7.11 | 7.30 | 7.30 |
| EC [mS∙cm^-1^] | 1.37 | 0.94 | 1.06 | 0.98 | 0.95 |
| BOD_5_ [mg∙L^-1^] | 430.00 | 270.00 | 235.00 | 85.00 | 15.00 |
| COD [mg∙L^-1^] | 683.00 | 423.00 | 369.00 | 143.00 | 81.00 |
| COD/BOD_5_ [-] | 1.59 | 1.56 | 1.57 | 1.68 | 5.40 |
| Orthophosphates [mgP∙L^-1^] | 9.50 | 6.20 | 1.70 | 0.50 | 0.20 |
| Total P [mgP∙L^-1^] | 14.40 | 8.70 | 5.10 | 1.90 | 0.65 |
| Ammonia nitrogen [mgN∙L^-1^] | 44.70 | 12.60 | 8.80 | 1.80 | 0.80 |
| Nitrates [mgN∙L^-1^] | 0.82 | 0.60 | 0.50 | 6.00 | 1.00 |
| Total N [mgN∙L^-1^] | 61.00 | 20.50 | 17.80 | 10.30 | 1.90 |
| TSS [mg∙L^-1^] | 387.00 | 286.00 | 197.00 | 77.00 | 35.00 |

Table S5. Average physicochemical indicators of municipal wastewater treated in WWTP B (PE = 35.000) with activated sludge (SBR technology) used in the study

| Parameter | Wastewater | | | |
| --- | --- | --- | --- | --- |
|  | influent | collected from retention chamber (after mechanical treatment) | from SBR (after 190 minutes of aeration) | from SBR (end of sedimentation) |
| pH | 6.80 | 6.90 | 7.00 | 7.00 |
| EC [mS∙cm^-1^] | 2.07 | 1.48 | 1.00 | 0.79 |
| BOD_5_ [mg∙L^-1^] | 1000.00 | 820.00 | 42.00 | 13.00 |
| COD [mg∙L^-1^] | 1650.00 | 1068.00 | 168.00 | 108.00 |
| COD/BOD_5_ [-] | 1.65 | 1.30 | 4.00 | 8.31 |
| Orthophosphates [mgP∙L^-1^] | 24.50 | 23.00 | 1.20 | 0.30 |
| Total P [mgP∙L^-1^] | 11.80 | 13.20 | 7.70 | 1.0 |
| Ammonia nitrogen [mgN∙L^-1^] | 51.80 | 56.90 | 5.10 | 1.30 |
| Nitrates [mgN∙L^-1^] | 0.60 | 0.70 | 11.00 | 0.20 |
| Total N [mgN∙L^-1^] | 71.00 | 59.70 | 17.10 | 2.20 |
| TSS [mg∙L^-1^] | 659.00 | 1113.00 | 185.00 | 32.00 |

Table S6. Average physicochemical indicators of industrial (dairy) wastewater from considered MPP

| Parameter | Wastewater | |
| --- | --- | --- |
|  | Raw | After flotation |
| pH | 6.90 | 7.20 |
| EC [mS∙cm^-1^] | 2.30 | 2.10 |
| BOD_5_ [mg∙L^-1^] | 1340.00 | 748.00 |
| COD [mg∙L^-1^] | 2150.00 | 1225.00 |
| COD/BOD_5_ | 1.60 | 1.63 |
| Orthophosphates [mgP∙L^-1^] | 20.90 | 11.30 |
| Total phosphorus [mgP∙L^-1^] | 38.00 | 20.50 |
| Ammonia nitrogen [mgN∙L^-1^] | 21.00 | 15.00 |
| Nitrates [mgN∙L^-1^] | 3.20 | 17.00 |
| Total nitrogen [mgN∙L^-1^] | 50.00 | 46.00 |
| TSS [mg∙L^-1^] | 347.00 | 220.00 |

Table S7. Characteristics and GC-MS parameters of target benzotriazoles

| **Compound** | **Main applications** | **Structure** | ***CAS* number** | ***MW* (g/mol)** | ***RT** (min)** | ***LTPRI**  (HP-5MS)** | ***m/z**** |
| --- | --- | --- | --- | --- | --- | --- | --- |
| BT | Corrosion inhibitor, drug and detergent precursor, aviation deicer | 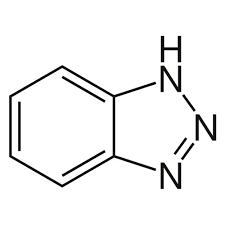 | 95-14-7 | 119.13 | 8.1±0.1 | 1389±1 | 133, 161, 119 |
| 4MBT | Anti-rust and copper corrosion  inhibitor | 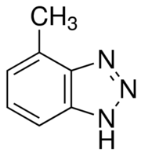 | 136-85-6 | 133.15 | 9.2±0.1 | 1476±1 | 104, 133, 175 |
| 5MBT | Anti-rust and copper corrosion  inhibitor | 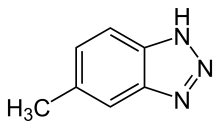 | 29878-31-7 | 133.15 | 9.5±0.1 | 1507±1 | 104, 146, 175 |
| 5ClBT | Drug and UV light absorbers  precursor | 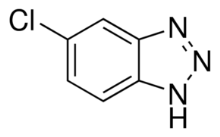 | 94-97-3 | 153.57 | 9.9±0.1  10.1±0.1 | 1540±1  1543±1 | 153, 195, 104 |
| UV326 | Ultraviolet light absorber, production of plastic and fabrics | 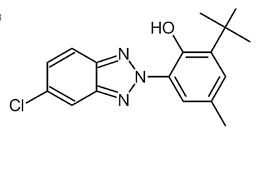 | 3846-71-7 | 315.80 | 16.0±0.1 | 2576±2 | 300, 315, 272 |
| UV329 | Ultraviolet absorber, production of plastic  and fabrics | 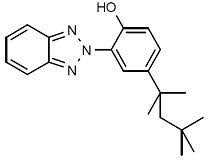 | 3147-75-9 | 323.43 | 16.4±0.1 | 2601±2 | 252, 323, 253 |

*-values determined for acetylated derivatives (except UV326); *CAS*–Chemical Abstract Service*, MW* – molar weight, *RT* – retention time, *LTPRI* – linear temperature programmed retention index^S1^; *m/z* - mass-to-charge ratio for quantification and identification ions; underlined m/z values for quantification ions.

S1. Van Den Dool, H. & Kratz, P.D. A Generalization of the retention index system including linear temperature programmed gas—liquid partition chromatography. *J. Chromatogr.* **11**, 463-471 (1963).
